# How Inclusive Are Patient Decision Aids for People with Limited Health Literacy? An Analysis of Understandability Criteria and the Communication about Options and Probabilities

**DOI:** 10.1177/0272989X241302288

**Published:** 2024-12-14

**Authors:** Romy Richter, Jesse Jansen, Josine van der Kraan, Wais Abbaspoor, Iris Bongaerts, Fleur Pouwels, Celine Vilters, Jany Rademakers, Trudy van der Weijden

**Affiliations:** Department of Family Medicine, Care and Public Health Research Institute (CAPHRI), Maastricht University, Maastricht, the Netherlands; Department of Family Medicine, Care and Public Health Research Institute (CAPHRI), Maastricht University, Maastricht, the Netherlands; Netherlands Patients Federation, Utrecht, the Netherlands; Department of Family Medicine, Care and Public Health Research Institute (CAPHRI), Maastricht University, Maastricht, the Netherlands; Department of Family Medicine, Care and Public Health Research Institute (CAPHRI), Maastricht University, Maastricht, the Netherlands; Department of Family Medicine, Care and Public Health Research Institute (CAPHRI), Maastricht University, Maastricht, the Netherlands; Department of Family Medicine, Care and Public Health Research Institute (CAPHRI), Maastricht University, Maastricht, the Netherlands; Department of Family Medicine, Care and Public Health Research Institute (CAPHRI), Maastricht University, Maastricht, the Netherlands; Netherlands Institute for Health Services Research (Nivel), Utrecht, the Netherlands; Department of Family Medicine, Care and Public Health Research Institute (CAPHRI), Maastricht University, Maastricht, the Netherlands

**Keywords:** shared decision making, risk communication, health literacy, patient decision aids

## Abstract

**Objective:**

Patient decision aids (PtDAs) can support shared decision making. We aimed to explore how inclusive PtDAs are for people with limited health literacy (LHL) by analyzing 1) the understandability of PtDAs using established criteria, 2) how options and probabilities of outcomes are communicated, and 3) the extent to which risk communication (RC) guidelines are followed.

**Methods:**

In a descriptive document analysis, we analyzed Dutch PtDAs available in 2021 that met the International Patient Decision Aid Standards. We developed and pilot tested a data extraction form based on key RC and health literacy literature.

**Results:**

Most PtDAs (151/198) met most of the understandability criteria on layout (7–8 out of 8 items) such as font size but not on content aspects (121/198 PtDAs scored 5–7 out of 12 items) such as defining medical terms. Only 31 of 198 PtDAs used a short and simple sentence structure. Most PtDAs presented 2 to 4 treatment options. Many followed RC recommendations such as the use of numerical RC strategies such as percentages or natural frequencies (160/198) and visual formats such as icon arrays (91/198). Only 10 used neutral framing (10/198). When presented, uncertainty was presented verbally (134/198) or in ranges (58/198). Four PtDAs were co-created together with patients with LHL and used only verbal RC or no RC.

**Conclusion:**

Most PtDAs met most of the understandability criteria on layout, but content aspects and adherence to RC strategies can be improved. Many PtDAs used long sentences and mostly verbal RC and are therefore likely to be inappropriate for patients with LHL. Further research is needed on PtDA characteristics and RC strategies suitable for people with LHL.

**Highlights:**

- Despite meeting most criteria for understandability, many of the Dutch PtDAs use long sentences, which likely impede comprehension for patients with LHL.
- Most of the Dutch PtDAs follow established recommendations for risk communication, with room for improvement for some strategies such as framing and a clear reference to the time frame.
- Overall, more research is needed to tailor PtDAs to the needs of people with LHL.

Shared decision making (SDM) and related decision support tools such as patient decision aids (PtDAs) have become central to patient-centered care.^1,2^ SDM is particularly indicated for preference-sensitive health care decisions where there is no single best option from a clinical perspective. A key step in SDM is to provide trustworthy information about treatment, diagnostic, or screening options through “option talk.” Option talk involves informing patients about medical options including the pros and cons and the likelihood of them occurring, known as risk communication (RC).^3^

PtDAs can support option talk and RC by making the options explicit and by clearly presenting probabilities of the potential benefits and harms of each option.^4,5^ Systematic research shows improved knowledge, more realistic expectations, and increased involvement in decision making in patients who use PtDAs.^1,2^ A meta-analysis reported positive effects of PtDAs for people with limited health literacy (LHL) and showed a greater benefit for this disadvantaged group compared with those with higher literacy, education, or socioeconomic status.^6^ In terms of RC, research shows that visualization of options and probabilities improves understanding and decision making, in particular for patients with LHL.^7–10^ Risk can be communicated verbally, numerically, and visually.^11–13^ It is known that communicating risk in verbal terms only (e.g., “your risk is low”) leads to ambiguity in interpretation since people can perceive the risk differently.^14,15^ Numerical RC can be applied through percentages or natural frequencies (e.g., 20% of women who smoke will have a heart attack or 20 of 100 women who smoke will have a heart attack [fictional data]), whereby a clear reference class should be stated (e.g., 20% of women who smoke will have a heart attack in 10 y).^16,17^ Visual RC concerns graphical displays of risks (e.g., icon arrays, bar charts, or risk ladders).^7,18^ Risk information can be framed positively or negatively, for example, an 80% chance of survival versus a 20% chance of dying. Choice of framing can influence risk perception and decision making (e.g., by perceiving the risk as less risky when positively framed and riskier when negatively framed).^11,19^ Efficient ways to communicate uncertainty are still under debate, and there is little guidance on best practice approaches.^19–21^

Health literacy entails people’s knowledge, motivation, and competency to access, understand, appraise, and apply health information order to make judgments and decisions concerning their health.^22^ People with LHL are more likely to have a poor understanding of their diagnostic, screening, or treatment options and are more likely to experience decisional uncertainty and regret.^23,24^ Overall, research shows that SDM has benefits for patients with LHL such as increased participation, increased knowledge, informed choice, decision self-efficacy, and reduced decisional conflict.^1,25^ However, despite the proven benefits of SDM, involving people with LHL remains challenging. Patient-facing materials often fail to communicate information clearly and understandably, hindering active participation in decision making and limiting their understanding of risk information.^26–28^ Therefore, option talk and RC are considered challenging in clinical practice.^29,30^ Some might argue whether people with LHL want to take part in SDM at all, as it is commonly believed that patients with LHL prefer to take a passive role in the decision-making process,^29,31^ although robust evidence is limited.^32^ However, research has shown that all patients, including those with LHL, want to be involved in decisions concerning their health and care, but they need clear and understandable information and communication to do so.^24^

As a patient’s ability to use PtDAs effectively is determined not only by their health literacy but also by the quality and suitability of the PtDA, it is vital that PtDA developers ensure that their tools are well designed and understandable to all.^33,34^ The International Patient Decision Aid Standards (IPDAS) Collaboration and other research suggests that the needs of people with LHL should receive special consideration during the PtDA development process.^23,34–36^ While most PtDA assessment studies focus on readability and understandability criteria, this study specifically aims to focus on option talk and RC within Dutch PtDAs. We aimed to explore how inclusive PtDAs are for people with LHL by analyzing 1) the understandability of PtDAs using established criteria, 2) how options and probabilities of outcomes are communicated, and 3) the extent to which RC guidelines are followed.

## Methods

### Study Design and Identification of PtDAs

This cross-sectional document analysis of Dutch PtDAs available in 2021 is primarily based on a list of 264 PtDAs, compiled by the Netherlands Patients Federation. In addition, a search was performed on PtDA platforms resulting in a total of *N* = 290 PtDAs for potential inclusion. For the additional search, we first screened the Web site Med-Decs, which 1) collects PtDAs on several topics and 2) provides an overview of other Web sites that present PtDAs. Second, other Web sites were screened (deelkunde.nl, kanker.nl, and thuisarts.nl). Third, the Web sites of the 2 main developers of PtDAs in the Netherlands (Patient+ and Zorgkeuzelab) were examined. The Medical Ethical Review Board (METC azM/UM) granted approval for our study (2021-2556).

**Figure 1:**
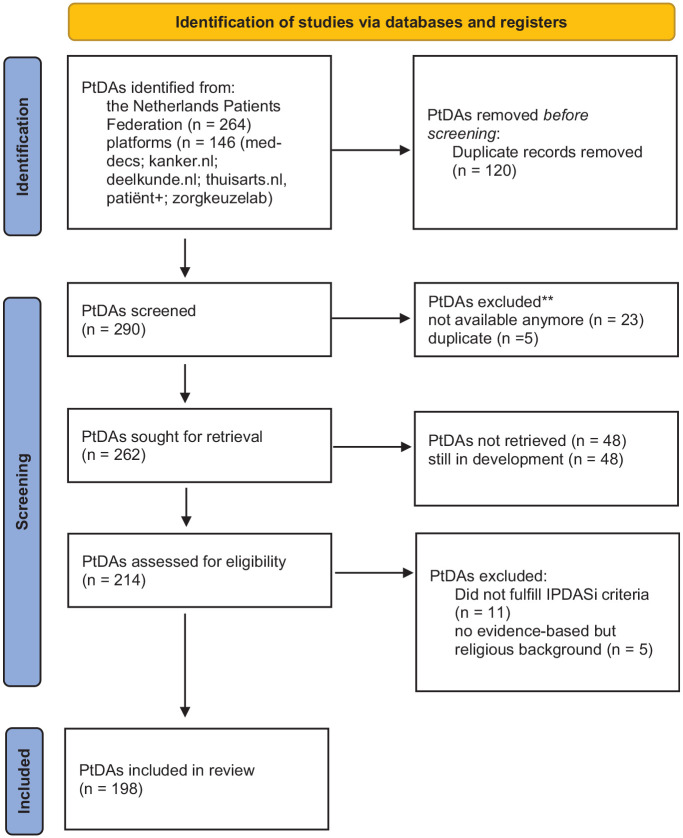
Flow chart.

Available and accessible PtDAs were included if they met the IPDASi criteria (Table 1 and Figure 1). Two researchers (I.B./F.P./C.V./W.A./J.L./M.J.) performed the screening for inclusion and exclusion independently. Subsequently, those researchers worked in pairs until consensus was reached. If no consensus was reached, a third independent researcher (R.R.) was consulted.

**Table 1:** Inclusion and Exclusion Criteria for PtDAs

Inclusion Criteria	Exclusion Criteria
• PtDAs accessible online • Available in Dutch • Meeting the International Patient Decision Aid Standards instrument (IPDASi) criteria^37^ ○ Describes health condition or problem for which index decision is required ○ Explicitly states decision under consideration ○ Describes the options available for the index decision ○ Describes the positive features of each option ○ Describes the negative features of each option ○ Describes the features of options to help patients imagine the physical, social and/or psychological effect	• Not available • Still in development • Do not fulfill IPDASi criteria • Not evidence-based but religious background

### Data Extraction Process and Variables

Dutch PtDAs were evaluated using published recommendations for understandability criteria for people with LHL as well as for RC strategies for the general population and specifically for patients with LHL. In our study, understandability captures general criteria on the content of PtDA information (e.g., the use of abbreviations, medical terms, or long sentences) and its presentation, which is referred to as *layout* (e.g., the font size, text structure, or use of sharp contrast). A data extraction sheet was developed in Excel containing qualitative and quantitative variables (Appendix 1). The data extraction sheet was independently piloted by 4 researchers each applying assets of 5 randomly chosen PtDAs. Based on this, the data extraction sheet was refined and edited. For all sections, data were collected either through dichotomy (e.g., yes/no present) or nominal values (various discriminating categories) or by narrative descriptions.

Each PtDA was extracted by 2 researchers independently (I.B./F.P./W.A./C.V./M.J./J.L.) and discussed in regular consensus meetings to resolve any discrepancies. If consensus could not be reached, the researchers consulted a third researcher (R.R.). Since consensus between researchers was obtained through agreement, a quantitative calculation of kappa was not performed.

The following data were extracted: 1) general PtDA characteristics; 2) understandability criteria related to clarity of PtDA information (layout and content); 3) description of options and treatment burden, as well as type of outcomes such as benefits and harms; 4) type of RC applied in PtDAs such as verbal, numerical, visual, and information on framing and uncertainty; and 5) compliance with recommended RC strategies. Verbal RC refers to written information about benefits and harms that is presented only descriptively in words, such as “a small chance of headache” rather than in numbers such as “10 out of 100 get headache.”

Section 1 of the data extraction sheet includes general characteristics of the PtDA such as topic, format, availability, and development of PtDAs; relation to national guidelines; and endorsement by organizations and representatives (Appendix 1).

Section 2 categorizes the understandability criteria for clarity of PtDA information (layout and content). They were based on the Patient Education Material Assessment Tool (PEMAT),^38^ the Suitability assessment of Material (SAM),^39^ and Dutch criteria for simple use of language (“Keurmerk Gewone taal”)^40^ (Appendix 2). SAM criteria have been applied to assess patient education materials in earlier studies related to health literacy.^41,42^ PEMAT is also developed to assess the understandability and actionability of patient education materials for consumers of varying levels of health literacy.^36,43^

Sections 3 and 4 of the data extraction sheet were based on earlier work of the research group.^29^ Risk information was labeled as *neutrally framed* if the same risk information was presented both positively and negatively framed in verbal terms. An icon array without explicitly verbal framing was not considered as neutrally framed. We tried to assess uncertainty in PtDAs in 2 potential ways. Communication of uncertainty could be in an indirect descriptive way in words such as *probably* or *maybe*. Another way was more directly descriptive such as, “You cannot predict in advance who will benefit from chemotherapy and who will not” or “Not everybody will get those side effects.” Another option was the use of ranges such as confidence intervals.

Section 5 was based on key RC literature.^11,17,29,44–47^ Most RC literature refers to the general population and not specifically to patients with LHL.^36^ In section 5, the following items were considered specifically relevant for people with LHL: for verbal RC items 2 and 3 in particular, for numerical RC items 2 to 4, and for visual RC, both items (Table 2).^3^

**Table 2:** Adherence to Evidence-Based RC Strategies (*N* = 198)^a^

Item	Yes	No
Verbal RC		
Use verbal RC	198	—
Avoid use of verbal RC alone*	49	149^b^
Use neutral framing*	10	188
Contextualization: To make sense of the risk information, people may also be provided with comparisons to other risks or to risks of other people.	—	198
Numerical RC		
Avoid the use of NNT	198	—
Presenting risk probabilities*	160	38
As natural frequencies only*	106	54
Consistent denominator used for natural frequencies*	126 (of 153)	72 (of 153)
As natural frequencies and percentage	47	113
As percentage only	7	153
Use of absolute risk (reduction) instead of relative risk reduction	152	46
Reference class mentioned (e.g., 100 patients with diabetes mellitus type 2)	143	55
Time frame mentioned	68	115
If RR(R) is presented, report baseline risk (reduction)	2	196
Visual RC		
Use visual RC formats*	100	98
Icon array*	92	
Pie chart^c^	7	
Bar chart^d^	3	
Presenting numerical information in tables or icon arrays rather than only text*	107	91

NNT, number needed to treat; RC, risk communication; RR(R), Relative Risk (Reduction).

a “Yes” indicates adherence to the RC strategy. Items with Asterisk* represent RC strategies recommended for people with LHL based on McCaffrey et al. (2013)^23^ and a systematic literature review.^50^

b At least in one part of the PtDA information is presented in verbal terms alone (e.g., small risk, often), but it could be that in other parts of the PtDA numerical or visual RC was used as well.

c 5/7 used pie chart alone and 2/7 together with icon array or bar chart.

d 1/3 used bar chart alone and 2/3 together with icon array or pie chart.

A few items required adjustment during the data analysis process, which was documented. For example, after discussion in the research team, the table format was not considered a visual RC strategy since its only purpose was to structure the text as part of the layout to make it easier to read but not to visually communicate the risk message itself.

### Data Analysis

Data analysis was performed using descriptive analysis. Based on the scored items, categorical data were created in SPSS to conduct a quantitative analysis. Quantitative data were presented in the form of frequencies and qualitative data by means of narratives.

## Results

### Selection of PtDAs

A total of 198 PtDAs met the IPDASi criteria and were included for data extraction and analysis. The deadline for inclusion was December 18, 2021.

### General Characteristics of PtDAs

Most PtDAs (190/198) covered options about treatment, for example, the treatment options for knee arthrosis. A small number of PtDAs (7/198) focused on screening or diagnostic options such as PSA screening for prostate cancer or whether to do a CA125 blood test after treatment for ovarian carcinoma (see Table 3). For most topics, 1 PtDA existed. Breast cancer and pancreas carcinoma were the most prevalent topics, for which 11 and 7 PtDAs existed per topic, respectively.

**Table 3:** Topic of Patient Decision Aids (PtDAs) Structured according to the International Classification of Primary Care

Topic/Disease	
**A. General and unspecified**	
Anesthesiology (anesthesia: narcosis and postoperative pain relief for preoperative patients) (*n* = 3) Chronic pain in children	Treatment limits (reanimation, dialysis) Smoking cessation (*n* = 2)
**B. Blood, blood-forming organs, and immune mechanism**	
Acute myeloid leukemia Hemochromatosis	Leukemia chronic lymphatic
**D. Digestive**	
Colon carcinoma (*n* = 3) Carcinoma oropharyngeal (*n* = 2) Crohn’s disease Gallstone problems Inguinal hernia (*n* = 2) (primary care and secondary care)	Irritable bowel syndrome Esophagus carcinoma Pancreas carcinoma (*n* = 7) Reflux disease (*n* = 2)
**F. Eye**	
Cataract	Noninfectious uveitis
**H. Ear**	
Cochlea implant (*n* = 2) (children and adults)	Otitis media (*n* = 3)
**K. Circulatory**	
Abdominal aortic aneurysm Aortic stenosis Atrial fibrillation Cerebrovascular accident Carotis stenose Cardiac arrhythmia (implantable cardioverter defibrillator) *Familiar hypercholesterolemia*	Heart valve disease (*n* = 2) Immune thrombopenia Peripheral artery disease Periphery arterial vascular disease (*n* = 2) Therapy-resistant hypertension Thrombose and pulmonary embolism Varices (*n* = 2) Venous malformities
**L. Musculoskeletal**	
Arthrosis in the thumb Bursitis elbow Biceps tendon rupture Bone tumor Broken shoulder Carpal tunnel syndrome (*n* = 2) Dupuytren’s disease Ganglion cyst wrist Humerus fracture Lateral clavicle fracture Mallet finger met fracture Mucoid cyst Midshaft clavicle fracture	Olecranon fracture (*n* = 2) Osteoporosis Ruptured rotator cuff Retinaculum cyst Radial head fracture Radius fracture Scaphoid fracture Shoulder instability Tenovaginitis of De Quervain’s Trigger finger Tennis elbow Trauma: recovery following fracture Wrist fracture
**L. Musculoskeletal (lower limbs)**	
Achilles tendon rupture Cruciate ligament injury (*n* = 3)	Hip arthrosis (*n* = 3) Knee arthrosis (*n* = 2)
**N. Neurologic**	
Epilepsy (*n* = 3) Lower back hernia (*n* = 2) Parkinson disease (*n* = 3)	Spinal cord injury: colon problems (*n* = 2) Spinal cord injury: pain treatment
**P. Psychological**	
Bipolar disorder (*n* = 5)	*Dementia*
**R. Respiratory**	
Lung cancer (*n* = 4) Obstructive sleep apnea syndrome	Tonsillitis (children, adults) (*n* = 4)
**S. Skin**	
Eczema (*n* = 2) Hemangioma	Psoriasis (*n* = 2) Superficial basal cell carcinoma
**T. Endocrine, metabolic, and nutritional**	
Diabetes mellitus type 2 Diabetes mellitus type 1 (*n* = 2)	Obesity
**U. Urology**	
Bladder cancer (*n* = 3) Benign prostate hypertrophy Catheterization (spinal cord injury) Kidney stones Kidney failure (*n* = 5)	Overactive bladder Urinary symptoms man *Prostate carcinoma* (*n* = 3)^a^ Stress incontinence
**W/X/Y. Pregnancy, childbirth, family planning; female genital system and breast; male genital system**	
Anticonception (*n* = 5) Breast cancer (*n* = 11) Childbirth (*n* = 6) Desire to have children and malignancies (*n* = 2) Extremely premature birth Endometriosis (*n* = 2) GBS bacteria childbirth *HPV vaccination* Heavy menstrual blood loss (*n* = 2)	Hysterectomy (*n* = 2) In vitro fertilization (*n* = 2) Myoma Miscarriage *Ovarium carcinoma* (*n* = 5)^b^ Ovarium cyst *Prenatal testing* (*n* = 3) Transman (*n* = 2) Uterine prolapse (*n* = 3)

GBS, Group B streptococcus; HPV, human papillomavirus. Number in parentheses = quantity of PtDAs per topic if more than 1 available; italics = PtDAs on screening, diagnostics, and vaccination.

a One PtDA is on prostate-specific antigen screening.

b One PtDA is on CA125 blood test after treatment for ovarian carcinoma.

Most PtDAs were available as an interactive multipage Web-based tool (138/198). The remaining PtDAs (60/198) were 1 page or multipage pdf documents. Most PtDAs were available for free (145/198) and could be downloaded or printed (181/198).

Approximately half of the PtDAs were approved by a professional (99/198) or patient organization (118/198). More than half of the PtDAs (101/198) explicitly stated that they had involved patients in the development process, but only a few explicitly stated they involved patients with LHL (4/198). In 76 of 198 PtDAs, it was stated that they were linked to national clinical practice guidelines.

### Understandability Criteria for Clarity of Information (Layout and Content)

Overall, 8 items were related to criteria on layout aspects. Most PtDAs (151/198) adhered to 7 or 8 items out of 8 items on criteria for easy-to-read layout such as font size and font style (Table 4). The remaining PtDAs adhered to 5 or 6 items (46/198) out of 8 items, and 1 PtDA adhered to 3 items out of 8 items. An overview per PtDA can be found in Appendix 3. Most PtDAs used an easy-to-read font (198/198), informative headlines (197/198), and an overall sharp contrast (191/198). For further generic criteria on layout, see Table 4.

**Table 4:** Understandability Criteria for Layout and Content (*N* = 198)

Item	Yes^a^	No
Layout		
Is an easy-to-read font (no fancy script or lettering) used?	198	—
Are informative headlines used?	197	1
Is overall sharp contrast used (dark fonts on light backgrounds)?	191	7
Is the information presented in a logical sequence?	184	14
Is the text well-structured (e.g., with bullets, paragraphs, or text boxes)?	177	21
Are visual cues (e.g., arrows, boxes, larger font, highlighting) used to draw attention to key points?	165	33
Is the font size minimal 12 to 14 pt?	162	36
Is there white space (no dense text)?	147	51
Content		
Can the material be used without the user having to perform calculations?	198	—
Is the topic of the PtDA clear based on the cover page?	196	2
Does the material explain the purpose and benefits from the patient’s perspective?	195	3
Are medical terms used only to familiarize the audience with the terms?	185	13
Are there few difficult abbreviations used?	182	16
Is the use of words with double meaning, or difficult words, avoided?	150	48
Are visuals^b^ used to support the written information?	130	68
Is the material interactive?^c^	126	72
Are medical terms defined?	104	94
Are the visuals easy for readers to follow and understand?	90	108
Is a short and simple sentence structure used?^d^	31	167
Are key points reviewed at the end of each section/page?	17	181

PtDA, patient decision aid.

a “Yes” indicates easier to understand.

b Visuals include icon arrays, bar charts, pie charts, other graphs, and illustrations or videos.

c Encourages the patient to write, answer questions, ask questions, cut out forms, etc.

d Five lines per paragraph; fewer than 10 words per sentence.

Overall, 12 items were related to content aspects. No PtDA adhered to more than 10 items out of 12 items. Some PtDAs adhered to 8, 9, or 10 items (28/198) out of 12 items. Most PtDAs adhered to 5, 6, or 7 items (121/198) or to 0 to 4 items (49/198) out of 12 items. No PtDA expected the user to perform calculations. Most PtDAs presented the topic clearly on the cover page (196/198) and explained the material’s purpose and benefits from the patient’s perspective (195/198). Visuals such as graphs, general illustrations (e.g., illustration of a body part), and videos were used in 130 of 198 PtDAs and in 90 of 198 PtDAs; these visuals were easy to follow and understand. More than half of the PtDAs provided definitions of medical terms (104/198). Only a few PtDAs (31/198) used a short and simple sentence structure, and a few PtDAs summarized key points at the end of each section (17/198). For further generic criteria on content, see Table 4.

### Presentation of Options and Probabilities

Most PtDAs reported 2 to 4 options (2 options: 83/198; 3 options: 45/198; 4 options: 25/198). A smaller number showed 5 or more options (45/198). Most PtDAs (183/198) presented all options at once; only a few used a stepwise approach, in which users were presented with options in 2 (11/198 PtDAs) or 3 or more steps (4/198 PtDAs).

Most PtDAs provided probabilities for desired outcomes such as improvement in quality of life or relief of symptoms (161/198), cure (94/198), and survival (39/198), as well as for undesired outcomes such as treatment side effects (184/198); deterioration of symptoms, condition, or quality of life (149/198); or recurrence of the disease or event (82/198). Within most PtDAs, multiple desired and undesired outcomes were reported (164/198), often clearly separated, for example, in a table. Treatment or diagnostic burden was reported in most PtDAs (178/198).

Most PtDAs reported standardized risk estimates (196/198), whereas 2 reported personalized estimates regarding gender and age. In 9 PtDAs, risk estimates were personalized by stratifying by age, gender, or risk group and giving risk estimates for several patient types.

A small subsample of PtDAs (4/198) was developed in collaboration with patients with LHL. For these PtDA topics, more comprehensive PtDAs are also available. They covered the topics knee arthrosis treatment, hip arthrosis treatment, kidney failure treatment, and support for smoking cessation (https://www.thuisarts.nl/overzicht/keuzekaarten). In these PtDAs (printable PDF), all options were outlined on the first page with letters (*A* for the first option, *B* for the second option, and so forth) with an illustration for each option. On the subsequent pages, options were discussed in more detail with 1 option per page.^48^ RC was reduced to a minimum and when included presented verbally only (e.g., “problems with an infection in your knee may occur very rarely”).^49^

### RC Strategies

Table 2 shows how often RC strategies were used. The items with Asterisk* are RC strategies recommended for people with LHL.^23,50^

#### Verbal RC and framing

All PtDAs used verbal RC (198/198) using terms such as *small chance*. In 49 of 198 PtDAs, only verbal RC was used for at least 1 information section. None of the PtDAs used contextualization (e.g., “Your risk is smaller than the risk of having a car accident”). The most common way of framing risk information was a mixture of positive and negative framing (87/198). Several PtDAs used negative framing only (66/198). Neutral framing (the use of positive and negative framing for the same information) was seldomly used (10/198).

#### Numerical RC

For numerical RC, 10 items could be scored (Table 2). More than half of PtDAs adhered to 5 to 7 items (116/198). Some PtDAs adhered to 0 to 4 items (46/198) or 8 to 10 (36/198) items. Most PtDAs used numerical risk information (160/198) by using natural frequencies (106/198) or natural frequencies and percentage formats together (47/160) or as percentages only (7/198). These 2 strategies were also used together in some PtDAs. Mostly a consistent denominator was used for natural frequencies (123/198). PtDAs often mentioned the reference class (143/198) but less often the time frame used (e.g., in 10 y) (68/198). The PtDAs mainly used absolute risk (157/198) instead of relative risk (2/198), and none mentioned the number needed to treat. Readers did not have to calculate anything.

#### Visual RC

More than half of the PtDAs used visual RC (102/198) to present risk information, mostly icon arrays (92/198). Other graphs such as pie charts (7/198) or bar graphs (3/198) were rarely used. More than half of PtDAs (107/198) presented numerical information in tables or icon arrays rather than text alone. Six PtDAs showed other manners of communicating options and risk information. Three PtDAs navigated through all treatment options by showing the procedures in an animated body, for example, for treatment of abdominal aortic aneurysm. Another PtDA about lung cancer showed an overview of the likelihood of serious side effects of radiotherapy and surgery with dots translated in the legend to numerical RC (e.g., small dot = about 1 out of 100 patients and large dot = about 10 out of 100 patients). The dots immediately provided a gist about the prevalence of those serious side effects. One PtDA about heart valve replacement used icon arrays that could be adjusted based on gender and age.

#### Uncertainty

In 141 of 198 PtDAs, uncertainty was mentioned, mostly using words (134/198) such as *probably* or *maybe* or descriptions such as, “You cannot predict in advance who will benefit from chemotherapy and who will not,” or “Not everybody gets those side effects.” A few PtDAs provided a range (58/198).

## Discussion

We identified 198 Dutch PtDAs covering a range of mostly treatment decisions. Overall, most of the PtDAs appear to be easy to read, meeting most layout-related understandability criteria but showing lower adherence to content criteria. Almost half of PtDAs did not define medical terms clearly, and 82% of the PtDAs used long sentences, making them more difficult for people with LHL to read and understand. Fifty-one percent of PtDAS were developed with patients, and 4 of these were co-designed with people with LHL. Most PtDAs showed 2 to 4 options, and the most reported outcomes included improved quality of life, reduced symptoms, and potential side effects of treatment. The PtDAs largely followed the recommended strategies for numerical RC, but a quarter of the PtDAs used verbal RC alone, even though this is known to lead to ambiguity in risk perception. Neutral framing (i.e., framing the same risk information both positively and negatively) was applied indirectly using icon arrays and was rarely made explicit verbally. One-sided framing of health information can influence risk perception and decision making, especially for people with LHL who are known to be suspectable to framing.^51^ Therefore, it is questionable whether the use of icon arrays next to written numerical RC is sufficient to avoid framing bias.

We still do not know much about which RC strategies work best for people with LHL as research with representative samples of people with LHL is still limited.^50^ The 4 PtDAs that were co-designed with people with LHL did not use any numerical RC strategies; rather, benefits and harms were presented as written verbal RC only (e.g., a small chance of headache). The effect of those PtDAs on smoking cessation, hip and knee arthroses, and kidney disease has not been evaluated yet.^52^ Most PtDAs in our sample used well-known option talk and RC strategies. Seven PtDAs in our sample showed other formats for option talk and RC such as an animated journey through the body or using different sizes of dots to display the magnitude of the risk involved. However, use of those innovative formats and evidence is still limited so that suitability for people with LHL remains questionable and more research would be needed. In our study, it seems that Dutch PtDAs overall perform well and seem useful for people with sufficient HL, which would approximately correspond to 75% of the Dutch population as approximately 25% of the Dutch population has LHL.^53^ The differences between the extensive verbal and numerical RC in the large body of classic PtDAs and the limited and only verbal RC in the small subset developed with people with LHL, which have not yet been evaluated in this group, illustrate the dilemma of ensuring the provision of sufficient detail to support informed decision making against preventing information overload.^54^ This raises the question of which format is best suited for people with LHL. It is questionable whether the solution is to develop only simplified PtDAs.^49^ On one hand, a universal precaution approach might be useful as shown by research, suggesting that PtDAS that are adapted to groups with lower health literacy can also be used for a more general audience, whereas extensive PtDAs with difficult text seem not suitable for people with LHL.^55^ On the other hand, several patients want detailed information on benefits and harms and demand more detail in PtDAs. A study comparing a long and short PtDA on prostate cancer treatment showed that both versions were considered useful and acceptable by patients, but the long version led to greater knowledge and informed choice, although the difference was small.^56^ However, the volume of information does not solely determine the suitability of a PtDA for people with LHL. Thoroughly applying health literacy principles in PtDAs will not only simplify complex information by reducing text volume but should also ensure that the content is meaningful and tailored to the patient’s needs, helping them understand how the information applies to their individual situation. Adopting the most useful PtDA approach also depends on the specific context of the decision. Both formats that were presented in the study appear to have advantages and disadvantages, and the appropriate use and usefulness of PtDAs also depend on the context and if and how a health care professional guides the decision process in practice. While patient preferences for PtDAs and RC may play a role, further research is needed, as these preferences might aid decision making but may not necessarily determine the effectiveness of a given strategy.

Our findings could not easily be compared with other analyses of PtDAs as most studies focus on RC or on health literacy separately.^17,36^ A content analysis by Waters et al. 2021 reported on internet-based cancer risk assessment tools (*n* = 39 Web sites) and adherence to recommended RC strategies. It showed similar findings that most tools used difficult language and that more than half of the tools used visual RC and numerical RC.^57^ Another systematic evaluation of PtDAs on cardiovascular disease prevention medication did not look at RC particularly but used PEMAT assessment. This analysis showed that the PtDAs scored well on understandability (content, layout, and use of visual strategies) but not on actionability (breaking the information down into manageable steps and explaining how to use the graphs). Authors therefore concluded that CVD prevention PtDAs are not suitable for people with LHL.^58^

Another aspect that should be considered is that most PtDAs are available as Web-based tools, which could be problematic as health disparities have been shown for access and use of digital health information for people with LHL.^59,60^

### Strengths and Limitations

A strength of our study lies in the analysis of a comprehensive convenience sample of Dutch PtDAs from 2021. This sample includes PtDAs collected by the Dutch Patient Federation as well as those identified through our additional search. While we are confident that we have screened most available Dutch PtDAs, we acknowledge the possibility of missing some. Despite this, we did not encounter any missed PtDAs after completing our analysis. We believe that our sample provides a robust and representative example of the development of PtDAs within the country. A strength of our study is the detailed data extraction sheet that we developed based on key literature^17,23,44–47,61,62^ and pilot tested. This document analysis focused on understandability criteria and on the evaluation option talk and RC in Dutch PtDAs. A comprehensive assessment of other relevant categories, such as the actionability of PtDAS, was not feasible. Applying the full PEMAT and SAM assessment tools would have exceeded the scope and resources of this project. However, we carefully designed the data collection sheet by drawing from relevant existing tools and adding criteria for option talk and RC based on key literature. A limiting factor might be potential differences in interpretation between the several coders who performed the data extraction. This was prevented as much as possible by having a detailed manual describing each data extraction section, and conflicts were always discussed within the research team during regular consensus meetings. We were not able to involve patients in the evaluation of the PtDAs but conducted focus groups with patients with LHL to discuss preferences for PtDA communication strategies in a subsequent study. These results will be presented in a separate article.

### Practical Implications

To increase the involvement of all patients in SDM, it is essential to adapt PtDAs to the needs of patients with LHL. Therefore, people with LHL should be involved in the development and testing of PtDAs. Many PtDAS use long sentences and are therefore likely to be not clear and understandable for patients with LHL. At the minimum, developers of PtDAs and health care providers interested in using PtDAs should assess the quality of PtDAs, and increasingly, easy-to-use tools are developed to assess the understandability of texts.^63^ Many Dutch PtDAs seemed to follow RC recommendations, but some strategies such as the use of neutral framing, a clear time frame, and reduced use of only verbal RC should be reconsidered. Overall, more research is needed to specifically investigate clear and understandable formats for people with LHL. However, it should be considered that there may not be a “one size fits all” strategy. Different RC strategies can and should be chosen depending on the situation and patient preferences and ability.

## Conclusion

Many of the Dutch PtDAs included in this study met the recommended understandability criteria on layout, but there is still room for improvement on understandability criteria on content and in the utilization of RC strategies to support comprehensibility and subsequently medical decisions. For people with LHL, more research is needed on the strategies required for discussion of options and RC. Strategies that we know work (e.g., clear icon arrays) should be more widely implemented in guidelines and assessment tools to ensure a basis for well-designed PtDAs for all patients. In addition, patients (especially those with LHL) should be more involved in the development process of PtDAs.

## Supplemental Material

sj-docx-1-mdm-10.1177_0272989X241302288 – Supplemental material for How Inclusive Are Patient Decision Aids for People with Limited Health Literacy? An Analysis of Understandability Criteria and the Communication about Options and Probabilities Supplemental material, sj-docx-1-mdm-10.1177_0272989X241302288 for How Inclusive Are Patient Decision Aids for People with Limited Health Literacy? An Analysis of Understandability Criteria and the Communication about Options and Probabilities by Romy Richter, Jesse Jansen, Josine van der Kraan, Wais Abbaspoor, Iris Bongaerts, Fleur Pouwels, Celine Vilters, Jany Rademakers and Trudy van der Weijden in Medical Decision Making
