# Supplementary material for: How Inclusive Are Patient Decision Aids for People with Limited Health Literacy? An Analysis of Understandability Criteria and the Communication about Options and Probabilities
Source: Med Decis Making. 2024 Dec 14;45(2):143–55. doi: 10.1177/0272989X241302288 (PMC11736975; doi:10.1177/0272989X241302288)
Supplement: sj-docx-1-mdm-10.1177_0272989X241302288 – Supplemental material for How Inclusive Are Patient Decision Aids for People with Limited Health Literacy? An Analysis of Understandability Criteria and the Communication about Options and Probabilities [file sj-docx-1-mdm-10.1177_0272989X241302288.docx]

# Appendix 1 Data extraction sheet

**Section 1:** Identify characteristics of PtDA as reported in PtDA itself.

| **Question** | **Outcome measures^[[1]](#footnote-1)^** |
| --- | --- |
| Name of PtDA | Name |
| Topic (Disease) | Name disease (classified according to International Classification of Primary Care (ICPC)) |
| Decision to be made | Explain in words |
| Type/format of PtDA | Web-based or printed paper tool |
| Availability/Accessibility   1. Is it available for free 2. Can you access it without an account 3. Available in other languages than Dutch 4. Possible to download or print part of PtDA | Link   1. Yes/No 2. Yes/No 3. Yes/No if yes which languages 4. Yes |
| Development of PtDAs  Developer   1. Patients involved in development 2. Patient with low health literacy involved in development | Name Developer   1. Yes/No/Not mentioned 2. Yes/No/Not mentioned |
| Related to national guidelines. If yes how? | Yes/No/Not mentioned. |
| Approval by patient representatives/organization. If yes who? | Yes/No/Not mentioned. |
| Approval by professional representatives/professional body. If yes who? | Yes/No/Not mentioned. |

**Section 2:** Identify understandability criteria of PtDA information (layout and content)

| **Question** | **Outcome measures** |
| --- | --- |
| **Layout/design, text appearance/organization** |  |
| 1. Is the font size minimal 12-14 pt | Yes/No |
| 1. Is it an easy-to-read font (no fancy script or lettering) used | Yes/No |
| 1. Is overall sharp contrast used (dark fonts on light backgrounds) | Yes/No |
| 1. Is there much white space (no dense text)? | Yes/No |
| 1. Do the material’s sections use informative headlines? | Yes/no |
| 1. Is the text well-structured e.g. with bullets, paragraphs or text boxes? | Yes/No |
| 1. Does the material use visual cues (e.g., arrows, boxes, bullets, bold, larger font, highlighting) to draw attention to key points? | Yes/No |
| 1. Does the material present information in a logical sequence? | Yes/No |
| **Content** |  |
| Is the topic of the PtDA clear based on the cover page? | Yes/No |
| Does the material use common, everyday language?   1. Short and simple sentence structure (less than 8-10 words per sentence, 5 lines per paragraph) **less than 10 words per sentence** 2. Words with double meaning, difficult words? 3. Use of many difficult abbreviations? | 1. Yes/No 2. Yes/No 3. Yes/No |
| 1. Are medical terms only used to familiarize audience with the terms? 2. When medical terms are used, are they always defined? | 1. Yes/No 2. Yes/No |
| Is the material interactive (encourage the patient to write, answer questions, ask questions, cut out forms, etc)? | Yes/No |
| Does the material expect the user to perform calculations? | Yes/No. |
| Does the material explain the purpose and benefits from the patient’s perspective? | Yes/No. |
| Are key points reviewed at the end of each section/page | Yes/No |
| **Visuals** |  |
| Are visuals used to support the written information? | Yes/No |
| Are the visuals easy for readers to follow and understand? | Yes/No/NA |

**Section 3:** Identify the option talk and type(s) of outcomes/ risk that are discussed in the PtDA.

| **Question** | **Outcome** |
| --- | --- |
| How many options are displayed and how many levels? | Number of options and number of levels |
| Types of outcomes on benefits   1. Probability of survival 2. Probability of cure 3. Probability of improving quality of life, decrease of symptoms 4. Probability of additional positive side-effects 5. Other | 1. Yes/No 2. Yes/No 3. Yes/No 4. Yes/No 5. Yes/No |
| Type of undesirable outcomes (cons)   1. Risk of disease/event 2. Risk of recurrence of disease/event 3. Risk of deterioration of symptoms /condition/ quality of life 4. Risk of dying and end-of life 5. Risk of side effects of treatment 6. Risk of treatment or diagnostic burden 7. Risk of overtreatment 8. Other | 1. Yes/No 2. Yes/No 3. Yes/No 4. Yes/No 5. Yes/No 6. Yes/No 7. Yes/No 8. Yes/No |
| Are the risk estimates to be used for large groups of patients (standardized, generic estimates) and/or personalized (by use of individualized prediction rules, algorithms) | Standardized/personalized |

**Section 4:** Identify the type of risk communication strategies used as described in Figure 1.

| **Question** | **Outcome measure** |
| --- | --- |
| Are advantage and disadvantage clearly separated (e.g. in a table, different columns) | Yes/No |
| 1. Is there verbal risk communication 2. If yes, only words or also metaphors | 1. Yes/No. 2. Words/metaphors. |
| Framing positive or negative? | Positive framing/negative framing/ mixed framing /neutral framing/NA |
| Numerical risk communication:   1. use of percentage 2. natural frequency 3. AR 4. ARR 5. RR 6. RRR | 1. Yes/No/NA 2. Yes/No/NA 3. Yes/No/NA 4. Yes/No/NA 5. Yes/No/NA 6. Yes/No/NA |
| Does it expect the user to calculate something | Yes/No |
| Visual risk communication: use of diagrams, fact sheets schemes, drawings | Yes/No. If yes name it e.g. icon array |
| 1. Communication of uncertainty 2. Explanation of uncertainty to the patients | 1. Yes/no. If yes, words/range 2. Yes/No. |

**Section 5:** Identify adherence to recommendations on risk communication based on key literature, such as IPDAS criteria.

| **Question** | **Outcome measure^[[2]](#footnote-2)^** |
| --- | --- |
| Presenting risk probabilities   1. As frequencies (numerator/denominator) 2. As percentage formats | Yes/No/ NA   1. Yes/No 2. Yes/No |
| Use consistent format of presenting probabilities throughout PtDA | Yes/No/NA |
| Use consistent denominator of the risk estimates | Yes/No/NA |
| Mention the reference class, such as e.g., 100 patients with diabetes mellitus type II/… | Yes/No/NA |
| Mention time frame, such as e.g., in 10 years/ lifetime…. | Yes/No/NA |
| 1. Use of absolute risk (reduction) instead of relative risk reduction 2. If RR(R) is presented, report baseline risk (reduction) | 1. Yes/No/NA 2. Yes/No/NA |
| Avoid the use of NNT | Yes/No |
| Avoid the use of qualitative risk descriptors alone (such as high risk) | Yes/No/ NA |
| Use visual risk communication formats e.g., icon arrays, bar charts | Yes/No/ NA/ If yes explain e.g. icon array |
| Presenting numerical information in tables or pictographs rather than text | Yes/No/Both / NA/ None |
| Use of neutral framing | Yes/No/ NA |
| Contextualization: To make sense of the risk information, people may also be provided with comparisons to other risks or to risks of other people. | Yes/No/ NA |
| Any outside-the-box, innovative manner of communicating option, pros/cons/probabilities | Yes/No/ NA |

**Framework risk communication (RC)**

AR(R) = Absolute Risk (Reduction)

RR(R) = Relative Risk (Reduction)

NNT = Number Needed to Treat

Richter R, Giroldi E, Jansen J, van der Weijden T. A qualitative exploration of clinicians' strategies to communicate risks to patients in the complex reality of clinical practice. PLoS One. 2020 Aug 13;15(8):e0236751. doi: 10.1371/journal.pone.0236751.

# Appendix 2 Sources of items in data extraction sheet

| Item |
| --- |
| Is the font size minimal 12-14 pt?  *SAM Layout and typography item b) typography* |
| Is an easy-to-read font (no fancy script or lettering) used?  *PEMAT item 13* |
| Is overall sharp contrast used (dark fonts on light backgrounds)?  *SAM Layout and typography item a) layout factors* |
| Is there white space (no dense text)?  *SAM Layout and typography item a) layout factors* |
| Are informative headlines used?  *PEMAT item 9* |
| Is the text well-structured e.g., with bullets, paragraphs or text boxes?  *SAM Layout and typography item b) typography* |
| Are visual cues (e.g., arrows, boxes, larger font, highlighting) used to draw attention to key points?  *PEMAT item 12* |
| Is the information presented in a logical sequence?  *PEMAT item 10* |
| Is the topic of the PtDA clear based on the cover?  *PEMAT Item 1* |
| Is a short and simple sentence structure used ^a^?  *SAM Layout and typography item a) layout factors and Keurmerk gewone taal* |
| Are words with double meaning, difficult words used?  *SAM Literacy demand item (c) Vocabulary uses common words* |
| Are many difficult abbreviations used?  *SAM Literacy demand item (c) Vocabulary uses common words* |
| Are medical terms only used to familiarize the audience with the terms?  *SAM Literacy demand item (c) Vocabulary uses common words and PEMAT item 4* |
| Are medical terms defined?  *SAM Literacy demand item (c) Vocabulary uses common words* |
| Is the material interactive ^b^?  *PEMAT item 20 and item 22* |
| Does the material expect the user to perform calculations?  *PEMAT item 7* |
| Does the material explain the purpose and benefits from the patient’s perspective?  *PEMAT item 1* |
| Are visuals* used to support the written information?  *PEMAT item 15 and 16* |
| Are the visuals easy for readers to follow and understand?  *SAM Graphics item b) type of graphics and PEMAT item 17 and 18* |
| Are key points reviewed at the end of each section/page?  *SAM content item a and d* |

# Appendix 3 Overview understandability criteria on layout and content per PtDA

# Table Layout (8 items)

White 0-4 items 🡪 N= 1

Light Blue 5-6 items 🡪 N= 46

Middle blue 7-8 items 🡪 N= 151

| Num. PtDA | PtDA_topic | Is the font size minimal 12-14 pt? | Is an easy-to-read font (no fancy script or lettering) used? | Is overall sharp contrast used (dark fonts on light backgrounds)? | Is there white space (no dense text)? | Are informative headlines used? | Is the text well-structured e.g., with bullets, paragraphs or text boxes? | Are visual cues (e.g., arrows, boxes, larger font, highlighting) used to draw attention to key points? | Is the information presented in a logical sequence? |
| --- | --- | --- | --- | --- | --- | --- | --- | --- | --- |
| 1 | Abdominal aortic aneurysm | x | x | x | x | x | x | x | x |
| 3 | Achilles tendon rupture | x | x | x | x | x | x | x | x |
| 5 | Acute myeloid leukaemia | x | x | x | x | x | x | x | x |
| 11 | Anaesthesiology | x | x | x | x | x | x | x |  |
| 13 | Anticonception | x | x | x | x | x |  | x |  |
| 14 | Anticonception student | x | x | x |  | x |  | x |  |
| 15 | Anticonception general |  | x |  | x | x | x | x | x |
| 16 | Anticonception without hormons |  | x |  | x | x | x | x | x |
| 17 | Anticonception after birth |  | x |  | x | x | x | x | x |
| 19 | Aortic stenosis | x | x | x | x | x | x |  | x |
| 20 | Arthrosis in the thumb | x | x | x | x | x | x | x | x |
| 21 | Hip arthrosis | x | x | x | x | x | x | x | x |
| 22 | Hip arthrosis |  | x | x | x | x | x | x | x |
| 23 | Knee arthrosis | x | x | x | x | x | x | x | x |
| 24 | Knee arthrosis |  | x | x |  | x | x | x | x |
| 26 | Atrial fibrillation | x | x | x | x | x |  | x | x |
| 27 | Uterine prolapse | x | x | x | x | x | x | x | x |
| 28 | Uterine prolapse | x | x | x | x | x | x | x | x |
| 29 | Operation Uterine prolapse | x | x | x | x | x | x | x | x |
| 30 | Bariatrie | x | x | x | x | x | x | x | x |
| 31 | Superficial basal cell carcinoma | x | x | x | x | x | x | x | x |
| 32 | Treatment limits (Reanimation, Dialysis) | x | x | x | x | x | x | x | x |
| 34 | Childbirth after earlier Caesarean section | x | x | x |  | x | x | x | x |
| 35 | Childbirth after earlier Caesarean section | x | x | x | x | x | x | x | x |
| 36 | Childbirth after earlier Caesarean section | x | x | x | x | x | x | x | x |
| 40 | Childbirth: breech birth or Caesarean section |  | X | x |  | x | x | x | x |
| 41 | Childbirth: breech birth | x | x | x | x | x | x | x | x |
| 42 | Childbirth: breech birth | x | x | x | x | x | x | x | x |
| 43 | Biceps tendon rupture | x | x | x | x | x | x | x | x |
| 44 | Bipolar disorder | x | x | x | x | x | x |  | x |
| 45 | Behandelmogelijkheden met psychologische & psychosociale therapie | x | x | x |  | x | x |  | x |
| 46 | Behandeling met medicijnen | x | x | x |  | x | x |  | x |
| 47 | Hulpmiddelen voor herstel, participatie & re-integratie | x | x | x |  | x | x |  | x |
| 48 | Hulpmiddelen om meer grip op jouw situatie te krijgen | x | x | x |  | x | x |  | x |
| 49 | Bladder cancer |  | x | x | x | x | x | x |  |
| 50 | Bladder cancer (CIS) |  | x | x |  | x | x | x |  |
| 51 | Keuzehulp stoma of vervangblaas | x | x | x | x | x | x | x |  |
| 52 | Catheterisation (Spinal cord injury) | x | x | x | x | x | x | x | x |
| 54 | Atrial fibrillation | x | x | x | x | x | x | x | x |
| 55 | Breast cancer | x | x | x | x | x | x | x | x |
| 56 | Breast cancer | x | x | x | x | x | x | x | x |
| 57 | Breast cancer | x | x | x | x | x |  | x | x |
| 58 | Breast cancer | x | x | x | x | x | x | x | x |
| 59 | Breast cancer | x | x | x | x | x | x | x | x |
| 60 | Breast cancer | x | x | x | x | x | x | x | x |
| 61 | Breast cancer: breast reconstruction | x | x | x | x | x | x | x | x |
| 62 | Breast cancer: breast reconstruction | x | x | x | x | x | x | x | x |
| 65 | Breast cancer: palliative care | x | x | x | x | x | x | x | x |
| 66 | Breast cancer BRASA radiotherapy shared decision making | x | x | x | x | x | x | x | x |
| 68 | Breast cancer hormone therapy |  | x | x |  | x | x | x | x |
| 69 | Maligne Bone tumour children | x | x | x |  | x |  | x | x |
| 70 | Broken shoulder | x | x | x | x | x | x | x | x |
| 71 | Benign prostate Hypertrophy | x | x | x | x | x |  | x |  |
| 73 | Carotis stenose | x | x | x | x | x | x | x | x |
| 75 | Carpal Tunnel Syndrome |  | x | x | x | x | x | x | x |
| 76 | Carpal Tunnel Syndrome | x | x | x | x | x | x | x | x |
| 77 | Chronische pijn bij tieners | x | x | x | x | x | x | x | x |
| 78 | Cochlea Implant adults | x | x | x | x | x |  | x | x |
| 79 | Cochlea Implant children | x | x | x | x | x |  | x | x |
| 81 | Crohn´s disease | x | x | x | x | x | x | x | x |
| 84 | Cerebro Vascular Accident | x | x | x |  | x | x |  | x |
| 87 | Diabetes Mellitus type II | x | x | x | x | x |  | x | x |
| 88 | Diabetes Mellitus type I |  | x | x | x | x | x | x | x |
| 89 | Diabetes Mellitus type II |  | x |  | x | x | x | x | x |
| 91 | Dikkerdarmkanker | x | x | x | x | x | x | x | x |
| 94 | Hoog-risico stadium 2 darmkanker | x | x | x | x | x | x | x | x |
| 96 | Colon carcinoma palliative treatment | x | x | x | x | x | x | x | x |
| 97 | Dupuytren´s Disease | x | x | x | x | x | x | x | x |
| 98 | Spinal cord injury: colon problems | x | x | x | x | x | x |  | x |
| 99 | Spinal cord injury: colon problems | x | x | x |  | x | x |  | x |
| 100 | Spinal cord injury: pain treatment | x | x | x |  | x | x |  | x |
| 101 | Eczema |  | x | x | x | x | x | x | x |
| 102 | Eczema |  | x | x | x | x | x | x | x |
| 103 | Hysterectomy | x | x | x |  | x | x | x | x |
| 104 | Olecranon fracture | x | x | x | x | x | x |  | x |
| 105 | Olecranon fracture above 65 years | x | x | x | x | x | x |  | x |
| 106 | Bursitis elbow | x | x | x | x | x | x | x | x |
| 108 | Endometriose | x | x | x | x | x | x |  | x |
| 110 | Endometriose fertility | x | x | x | x | x | x | x | x |
| 111 | Epilepsy | x | x | x | x | x | x |  | x |
| 112 | Epilepsy | x | x | x | x | x | x |  | x |
| 113 | Epilepsy | x | x | x | x | x | x |  | x |
| 116 | Gallstone problems | x | x | x | x | x | x | x | x |
| 117 | GBS bacteria childbirth |  | x | x | x | x | x | x | x |
| 124 | Heart valve disease | x | x | x |  | x | x |  | x |
| 125 | Heart valve disease | x | x | x | x | x | x |  | x |
| 126 | Haemangioma | x | x | x | x | x |  | x | x |
| 127 | Hemochromatosis |  | x | x |  | x | x | x | x |
| 128 | Carcinoma oropharyngeal | x | x | x | x | x | x | x | x |
| 129 | HPV vaccination |  | x |  |  | x |  | x |  |
| 130 | Familiar hypercholesterolemia |  | x | x |  | x | x | x | x |
| 131 | Hypertension |  | x | x |  | x | x | x | x |
| 132 | Cardiac arrhythmia (Implantable Cardioverter Defibrillator ICD) | x | x | x | x | x |  | x | x |
| 134 | Immune thrombopenia | x | x | x | x | x | x | x | x |
| 135 | In-Vitro-Fertilisation | x | x | x | x | x | x | x | x |
| 136 | In-Vitro-Fertilisation: 1 or 2 embryos | x | x | x |  | x | x | x | x |
| 137 | Child-wish and malignancies | x | x | x |  | x | x |  | x |
| 138 | Child-wish and malignancies | x | x | x |  | x | x | x | x |
| 139 | Tonsillitis children | x | x | x |  | x | x |  | x |
| 140 | Tonsillitis adults | x | x | x | x | x | x |  | x |
| 141 | Tonsillitis | x | x | x | x | x | x | x | x |
| 142 | Tonsillitis children |  | x | x | x | x | x | x | x |
| 146 | Lower back hernia | x | x | x | x | x | x | x | x |
| 147 | Lower back hernia |  | x | x |  | x | x | x | x |
| 148 | Leukaemia chronic lymphatic | x | x | x | x | x | x | x | x |
| 150 | Inguinal hernia secondary care | x | x | x | x | x | x | x | x |
| 151 | Inguinal hernia primary care | x | x | x | x | x | x | x | x |
| 152 | Lung cancer | x | x | x | x | x | x | x | x |
| 153 | Lung cancer | x | x | x |  | x | x | x | x |
| 156 | Lung cancer (NSCLC-stadium 1 or 2a) | x | x | x | x | x | x | x | x |
| 157 | Lung cancer | x | x | x | x | x |  | x |  |
| 159 | Mallet finger with fracture | x | x | x | x | x | x |  | x |
| 161 | Cruciate ligament injury | x | x | x | x | x | x | x | x |
| 162 | Heavy menstrual blood loss | x | x | x | x | x | x | x | x |
| 163 | Menstruation |  | x | x |  | x | x | x | x |
| 164 | Otitis media |  | x | x | x | x | x | x | x |
| 165 | Otitis media | x | x | x | x | x | x | x | x |
| 166 | Otitis media | x | x | x | x | x | x | x | x |
| 168 | Miscarriage | x | x | x | x | x | x | x | x |
| 172 | Mucoid cyste | x | x | x | x | x | x |  | x |
| 173 | Myoma | x | x | x | x | x | x | x | x |
| 174 | Kidney failure | x | x | x | x | x |  | x | x |
| 175 | Kidney failure |  | x | x |  | x | x | x | x |
| 176 | Kidney failure | x | x | x | x | x | x | x | x |
| 177 | Kidney failure |  | x | x |  | x | x | x | x |
| 179 | Kidney stones | x | x | x | x | x | x | x | x |
| 181 | Obstructive Sleep apnoea Syndrome |  | x | x |  | x | x | x | x |
| 182 | Osteoporosis | x | x | x | x | x | x | x | x |
| 184 | Ovarium cyst |  | x | x |  | x | x | x | x |
| 185 | CA125 after treatment ovarium carcinoma |  | x | x | x | x | x | x | x |
| 187 | Ovarium carcinoma | x | x | x |  | x | x | x | x |
| 188 | Hysterectomy | x | x | x | x | x | x | x | x |
| 189 | Ovarium carcinoma |  | x | x |  | x | x | x | x |
| 190 | Ovarium carcinoma |  | x | x |  | x | x | x | x |
| 191 | Overactive bladder | x | x | x | x | x |  | x | x |
| 196 | Pancreas carcinoma | x | x | x |  | x | x |  | x |
| 199 | Parkinson Disease |  | x | x |  | x | x | x | x |
| 200 | Parkinson Disease | x | x | x |  | x |  | x |  |
| 201 | Parkinson Disease | x | x | x |  | x | x | x | x |
| 202 | Tenovaginitis of De Quervain´s | x | x | x | x | x | x | x | x |
| 203 | Periphery Arterial disease | x | x | x | x | x | x | x | x |
| 204 | Periphery Arterial disease | x | x | x | x | x | x | x | x |
| 205 | Urinary symptoms man | x | x | x | x | x | x | x | x |
| 207 | Wrist fracture | x | x | x | x | x | x |  | x |
| 208 | Ganglion cyst wrist | x | x | x | x | x | x | x | x |
| 210 | Prenatal testing | x | x | x |  | x | x | x | x |
| 212 | Prenatal testing: 20 weeks echo | x | x | x | x | x |  | x |  |
| 213 | Prenatal testing: screening down syndrome | x | x | x | x | x |  | x |  |
| 214 | Irritable bowel syndrome | x | x | x | x | x | x | x | x |
| 216 | Prostate cancer | x | x | x |  | x |  | x | x |
| 219 | Prostate cancer | x | x | x | x | x | x | x | x |
| 220 | Prostate cancer CRPC | x | x | x | x | x | x | x | x |
| 221 | Prostate cancer | x | x | x | x | x | x | x | x |
| 223 | Prostate cancer | x | x | x | x | x | x | x | x |
| 224 | Prostate cancer | x | x | x | x | x | x | x | x |
| 225 | Psoriasis | x | x | x | x | x | x | x | x |
| 227 | Psoriasis severe 1 |  | x | x |  | x | x | x | x |
| 230 | Reflux disease | x | x | x | x | x | x | x | x |
| 231 | Reflux disease | x | x |  | x | x | x | x | x |
| 232 | Retinaculum cyst | x | x | x | x | x | x |  | x |
| 233 | Smoking cessation |  | x |  |  | x | x | x | x |
| 234 | Ruptured rotator cuff | x | x | x |  | x | x |  | x |
| 235 | Scaphoid fracture | x | x | x | x | x | x |  | x |
| 238 | Broken shoulder | x | x | x | x | x | x |  | x |
| 239 | Shoulder instability | x | x | x | x | x | x | x | x |
| 240 | Lateral clavicle fracture | x | x | x | x | x | x |  | x |
| 241 | Midshaft clavicle fracture | x | x | x | x | x | x |  | x |
| 243 | Oesophagus carcinoma |  | x | x |  | x | x | x | x |
| 244 | Olecranon fracture | x | x | x | x | x | x |  | x |
| 248 | Cataract | x | x | x | x | x | x | x | x |
| 249 | Stress incontinence | x | x | x | x | x |  | x | x |
| 250 | Carcinoma oropharyngeal | x | x | x | x | x | x | x | x |
| 251 | Tennis elbow | x | x | x | x | x | x | x | x |
| 253 | Transman operation | x | x | x | x | x | x | x |  |
| 254 | Transman fertility | x | x | x | x | x | x | x |  |
| 255 | Trauma: recovery following fracture | x | x | x |  | x | x | x | x |
| 256 | Trigger Finger | x | x | x | x | x | x | x | x |
| 257 | Thrombose and pulmonary embolism |  | x | x | x | x | x | x | x |
| 258 | Hysterectomy | x | x | x | x | x | x | x | x |
| 259 | Non-infectious uveitis | x | x | x | x | x | x | x | x |
| 260 | Varices | x | x | x | x | x | x | x | x |
| 261 | Varices 2 | x | x | x |  | x | x | x | x |
| 262 | Venous malformities | x | x | x |  | x |  | x | x |
| 263 | Anaesthesiology | x | x | x | x | x | x | x | x |
| 264 | Anaesthesiology | x | x | x | x | x | x | x | x |
| 266 | Cruciate ligament injury |  | x | x |  | x | x | x | x |
| 267 | Cruciate ligament injury | x | x | x | x | x | x | x | x |
| 268 | Extremely premature birth | x | x | x |  | x | x | x | x |
| 271 | Knee arthrosis | x | x | x | x | x | x | x | x |
| 274 | Varices | x | x | x | x |  | x | x | x |
| 275 | Periphery Arterial vascular disease | x | x | x |  | x | x | x | x |
| 276 | Hip arthrosis | x | x | x | x | x | x | x | x |
| 277 | Kidney failure | x | x | x | x | x | x | x | x |
| 278 | Smoking cessation | x | x | x | x | x | x | x | x |
| 280 | Dementia | x | x | x | x | x | x | x | x |
| 281 | Prostate carcinoma | x | x | x | x | x | x | x | x |
| 282 | Prostate carcinoma | x | x | x | x | x | x |  | x |
| 283 | Prostate carcinoma | x | x | x |  | x | x | x | x |

# Table - Content (12 items)

Light orange 0-4 items 🡪 N= 49

Middle orange 5-7 items 🡪 N= 121

Dark orange 8-10 items 🡪 N= 28

| PtDA | PtDA_Name | Is the topic of the PtDA clear based on the cover? | Is a short and simple sentence structure used ^a^? | Are words with double meaning, difficult words used? | Are many difficult abbreviations used? | Are medical terms only used to familiarize the audience with the terms? | Are medical terms defined? | Is the material interactive ^b^? | Does the material expect the user to perform calculations? | Does the material explain the purpose and benefits from the patient’s perspective? | Are key points reviewed at the end of each section/page? | Are visuals* used to support the written information? | Are the visuals easy for readers to follow and understand? |
| --- | --- | --- | --- | --- | --- | --- | --- | --- | --- | --- | --- | --- | --- |
| 1 | Abdominaal aorta aneurysma | x |  |  |  | x |  | x |  | x |  | x | x |
| 3 | Gescheurde Achillespees | x |  |  |  | x | x | x |  | x |  | x | x |
| 5 | AML | x |  |  |  | x | x | x |  | x | x |  |  |
| 11 | Anesthesiologie | x |  |  |  | x | x | x |  | x | x |  |  |
| 13 | Anticonceptie voor jou | x |  |  |  | x | x | x |  | x |  | x |  |
| 14 | Anticonceptie student | x |  |  | x |  |  | x |  | x |  |  |  |
| 15 | Anticonceptie algemeen | x |  |  |  | x |  |  |  | x |  |  |  |
| 16 | Anticonceptie zonder hormonen | x |  |  |  | x |  |  |  | x |  |  |  |
| 17 | Anticonceptie na de bevalling | x |  |  |  | x |  |  |  | x |  |  |  |
| 19 | Ernstig vernauwde aortaklep (aortaklepstenose) | x | x |  |  | x | x |  |  | x |  |  |  |
| 20 | Artrose van de duim | x |  |  |  | x |  | x |  | x |  | x | x |
| 21 | Artrose van de heup | x |  |  |  | x |  | x |  | x |  | x | x |
| 22 | Artrose van de heup 2 | x |  |  |  |  | x |  |  | x |  |  |  |
| 23 | Artrose van de knie | x |  |  |  | x | x | x |  | x |  | x | x |
| 24 | Artrose van de knie 2 | x |  | x |  | x |  |  |  | x |  |  |  |
| 26 | Atriumfibrilleren | x |  |  |  | x |  | x |  | x |  | x |  |
| 27 | Verzakking keuzehulp | x |  |  |  | x |  | x |  | x |  | x | x |
| 28 | Verzakking | x |  |  |  | x | x | x |  | x |  | x | x |
| 29 | Operatie bij een verzakking | x |  |  |  | x |  | x |  | x |  | x | x |
| 30 | Bariatrie | x | x |  |  | x | x | x |  | x | x | x | x |
| 31 | Basaalcelcarcinoom | x |  |  |  | x | x | x |  | x |  | x |  |
| 32 | Behandelgrenzen | x |  |  |  | x | x | x |  | x |  | x | x |
| 34 | Bevallen na een eerdere keizersnede (keuzehulp) | x |  |  |  | x | x | x |  | x |  | x | x |
| 35 | Bevallen na een eerdere keizersnede: samen kiezen met een keuzehulp | x |  |  |  | x |  | x |  | x |  |  |  |
| 36 | Keuzehulp baring na keizersnede | x |  |  |  | x | x | x |  | x |  |  |  |
| 40 | Uw kind ligt in een stuit: vaginale stuitbevalling of geplande keizersnede? | x |  |  |  | x |  |  |  | x |  |  |  |
| 41 | Keuzehulp: stuitbevalling | x |  |  |  | x |  | x |  | x |  | x | x |
| 42 | Keuzehulp: versie bij stuitligging | x |  |  |  | x | x | x |  | x |  | x | x |
| 43 | Bicepspees: gescheurd | x | x |  |  | x | x | x |  | x | x | x | x |
| 44 | Onderhouds-behandeling van een bipolaire stoornis | x | x | x |  | x |  | x |  | x | x | x |  |
| 45 | Behandelmogelijkheden met psychologische & psychosociale therapie | x |  |  |  | x | x |  |  | x |  |  |  |
| 46 | Behandeling met medicijnen | x |  |  | x | x | x |  |  | x |  |  |  |
| 47 | Hulpmiddelen voor herstel, participatie & re-integratie | x |  |  | x | x | x |  |  | x |  |  |  |
| 48 | Hulpmiddelen om meer grip op jouw situatie te krijgen | x |  |  | x | x | x |  |  | x |  |  |  |
| 49 | Blaaskanker | x |  |  |  | x |  |  |  | x |  |  |  |
| 50 | Blaaskanker carcinom in situ (CIS) | x |  |  |  | x |  |  |  | x |  |  |  |
| 51 | Keuzehulp stoma of vervangblaas | x |  |  |  | x |  | x |  | x |  | x | x |
| 52 | Katheterisatie | x | x |  |  | x |  | x |  | x | x | x | x |
| 54 | Boezemfibrilleren | x |  |  |  | x |  | x |  | x |  | x |  |
| 55 | Borstkanker Keuzehulp | x |  |  |  | x |  | x |  | x |  | x | x |
| 56 | Borstkanker | x |  |  |  | x | x | x |  | x |  | x | x |
| 57 | Aanvullende behandeling bij borstkanker | x |  |  |  | x | x | x |  | x |  | x | x |
| 58 | Aanvullende behandeling bij borstkanker | x |  |  |  | x | x | x |  | x |  | x | x |
| 59 | Aanvullende behandeling bij borstkanker | x |  |  |  | x | x | x |  | x |  | x | x |
| 60 | Aanvullende behandeling bij borstkanker | x |  |  |  | x | x | x |  | x |  |  |  |
| 61 | Borstrecontructie keuzehulp | x |  | x |  | x |  | x |  | x |  | x | x |
| 62 | Borstreconstructie | x |  |  |  | x | x | x |  | x |  | x | x |
| 65 | Keuzehulp voor palliatieve chemotherpaie: mammacarcinoom | x |  |  |  | x | x |  |  | x |  |  |  |
| 66 | BRASA (borstkanker radiotherapie samen beslissen) | x |  |  |  | x | x | x |  | x |  | x | x |
| 68 | Niet uitgezaaide hormoongevoelige borstkanker: wel of niet volgen van aanvullende hormoontherapie | x |  |  |  | x |  |  |  | x |  |  |  |
| 69 | Kwaadaardige (kinder)bottumoren (keuzehulp) | x |  |  |  | x | x | x |  | x |  | x | x |
| 70 | Bovenarm gebroken | x | x |  |  | x | x | x |  | x | x | x | x |
| 71 | BPH keuzehulp | x |  | x | x |  |  | x |  | x |  | x |  |
| 73 | Carotisstenose | x | x |  |  | x | x |  |  | x |  | x | x |
| 75 | Carpale tunnel syndroom | x |  |  |  | x |  |  |  | x |  |  |  |
| 76 | Carpale tunnel syndroom | x |  |  |  | x | x | x |  | x |  | x | x |
| 77 | Chronische pijn bij tieners | x |  |  |  | x | x | x |  | x |  | x | x |
| 78 | Cochlear implantaat voor volwassenen | x |  |  |  | x |  |  |  | x |  |  |  |
| 79 | Cochlear implantaat voor kinderen | x |  |  |  | x |  |  |  | x |  |  |  |
| 81 | Ziekte van crohn | x |  |  |  | x | x | x |  | x |  | x |  |
| 84 | CVA | x |  |  |  | x | x | x |  |  |  | x | x |
| 87 | Diabetes | x |  |  |  | x |  | x |  | x |  | x | x |
| 88 | Diabetes type 1 | x |  | x |  | x |  |  |  | x |  |  |  |
| 89 | Diabetes type 2 | x |  | x | x |  |  |  |  | x |  |  |  |
| 91 | Dikkerdarmkanker keuzehulp | x |  |  |  | x |  | x |  | x |  | x | x |
| 94 | Hoog-risico stadium 2 darmkanker | x |  |  |  | x |  | x |  | x |  |  |  |
| 96 | Keuzehulp voor palliatieve chemotherapie bij coloncarcinoom | x |  |  |  | x |  |  |  | x |  |  |  |
| 97 | Ziekte van Dupuytren | x |  |  |  | x |  | x |  | x |  | x |  |
| 98 | Darmproblemen bij dwarslaesie of caudalaesie | x |  |  |  | x |  |  |  | x |  |  |  |
| 99 | Blaaskatheterisatie bij dwarslaesie/caudalaesie | x |  |  |  | x |  |  |  | x |  |  |  |
| 100 | Neurogene pijnbehandeling bij dwarslaesie/caudalaesie | x |  |  |  | x |  |  |  | x |  |  |  |
| 101 | Eczeem, systemisch | x |  | x |  | x |  |  |  | x |  |  |  |
| 102 | Eczeem, lokaal | x |  |  |  | x |  |  |  | x |  |  |  |
| 103 | Keuzehulp: preventief eierstokverwijdering | x |  |  |  | x | x | x |  | x |  | x | x |
| 104 | Olecranon gebroken | x | x |  |  | x | x | x |  | x | x | x | x |
| 105 | Elleboog gebroken boven 65 jaar | x | x |  |  | x | x | x |  | x | x | x | x |
| 106 | Bursa inflammation elbow | x |  |  |  | x | x | x |  | x |  | x | x |
| 108 | Endometriose | x |  | x | x | x |  |  |  | x |  | x | x |
| 110 | Keuzehulp vruchtbaarheidsbehoud endometriose | x |  |  |  | x | x |  |  | x |  | x | x |
| 111 | Epilepsie: Moeilijk behandelbaar bij volwassenen | x | x | x |  | x |  |  |  | x |  |  |  |
| 112 | Epilepsie: nieuw gediagnosticeerd bij volwassenen | x | x | x |  | x | x |  |  | x |  |  |  |
| 113 | Epilepsie: partiele aanvallen bij volwassenen | x | x | x |  | x | x |  |  | x |  |  |  |
| 116 | Galsteenlijden | x |  |  |  | x | x | x |  | x |  | x | x |
| 117 | Draagster van de GBS-bacterie en geen risicofactoren, wel of geen antibiotica tijdens de bevalling (consultkaart) | x |  |  |  | x | x |  |  | x |  |  |  |
| 124 | Hartklepvervanging | x |  | x |  | x |  | x |  | x |  | x |  |
| 125 | Hartklepvervanging | x | x |  |  | x | x | x |  | x |  | x | x |
| 126 | Hemanchiomen | x |  | x |  |  |  | x |  | x |  | x |  |
| 127 | Hemochromatose | x |  | x |  | x | x |  |  | x |  |  |  |
| 128 | Hoofd-halskanker: passende nazorg | x | x |  |  | x | x | x |  | x |  |  |  |
| 129 | HPV vaccinatie | x |  |  | x | x | x | x |  | x |  | x |  |
| 130 | Hypercholesterolemie | x |  | x |  | x | x |  |  | x |  |  |  |
| 131 | Hypertension | x |  |  |  | x | x |  |  | x |  |  |  |
| 132 | ICD | x |  | x | x | x | x | x |  | x |  | x |  |
| 134 | Immuun trombopenie | x |  | x |  | x | x | x |  | x |  | x | x |
| 135 | IVF | x |  |  |  |  |  | x |  | x |  | x |  |
| 136 | IVF: 1 of 2 embryo's terugplaatsen | x |  |  |  | x |  | x |  | x | x |  |  |
| 137 | Kanker en kinderwens | x |  | x |  | x | x | x |  | x |  | x | x |
| 138 | Keuzehulp vruchtbaarheidsbehoud kanker | x |  |  |  | x | x |  |  | x |  | x | x |
| 139 | keelamandelen (onsteking) bij kinderen (<16jr) | x | x |  |  | x | x | x |  | x | x | x | x |
| 140 | keelamandelen bij volwassenen | x | x |  |  | x | x | x |  | x | x | x | x |
| 141 | Keelpijn | x | x |  |  | x | x | x |  | x |  | x | x |
| 142 | Keelamandelen kind 2 | x |  |  |  | x |  |  |  | x |  |  |  |
| 146 | Lage rughernia | x |  |  |  | x | x | x |  | x |  | x |  |
| 147 | Lage rughernia 2 | x |  | x |  | x |  |  |  | x |  |  |  |
| 148 | Chronische lymfatische leukemie | x |  |  |  | x | x |  |  | x |  | x | x |
| 150 | Liesbreuk tweede lijn | x |  |  |  | x | x | x |  | x |  | x |  |
| 151 | Liesbreuk eerste lijn | x |  |  |  | x | x | x |  | x | x | x |  |
| 152 | Patient decision aid: lungcancer | x |  | x | x | x |  | x |  | x | x | x | x |
| 153 | Longkanker keuzehulp | x |  |  |  | x |  | x |  |  |  | x | x |
| 156 | Keuzehulp: Longkanker (NSCLC stadium 1 of 2a) | x |  |  |  | x |  | x |  | x |  | x | x |
| 157 | Keuzehulp longkanker | x |  |  |  | x |  | x |  | x |  | x | x |
| 159 | Mallet finger with fracture | x | x |  |  | x | x | x |  | x |  | x | x |
| 161 | Meniscus laesie | x |  |  |  | x | x | x |  | x |  | x |  |
| 162 | Heavy menstrual blood loss | x |  |  |  | x | x | x |  | x |  | x | x |
| 163 | Heavy menstrual blood loss | x |  | x |  | x |  |  |  | x |  |  |  |
| 164 | Middenoorontsteking 2 | x |  | x |  | x |  |  |  | x |  |  |  |
| 165 | Middenoorontstekingen | x |  | x |  | x |  | x |  | x |  | x | x |
| 166 | Middenoor: vocht in | x | x |  |  | x |  | x |  | x |  | x | x |
| 168 | Miscarriage | x |  |  |  | x | x | x |  | x |  | x | x |
| 172 | Mucoid cyste | x | x |  |  | x | x | x |  | x |  | x | x |
| 173 | Myoma | x |  |  |  | x |  |  |  | x |  | x | x |
| 174 | Kidney failure | x |  | x | x | x | x | x |  | x |  | x |  |
| 175 | Kidney failure | x |  | x |  | x |  |  |  | x |  |  |  |
| 176 | Kidney failure | x |  |  |  | x |  | x |  | x |  | x | x |
| 177 | Kidney failure | x |  |  |  | x |  |  |  | x |  |  |  |
| 179 | Kidney stones | x |  | x |  | x | x | x |  | x |  | x |  |
| 181 | OSAS (slaap apneu) | x |  |  | x | x |  |  |  | x |  |  |  |
| 182 | Osteoporosis | x |  | x |  | x | x | x |  | x |  |  |  |
| 184 | Ovarium cyst | x |  |  |  | x |  |  |  | x |  |  |  |
| 185 | Argumentenkaart CA125 | x |  | x |  | x |  |  |  | x |  |  |  |
| 187 | Ovarium carcinoma | x |  | x | x | x |  | x |  | x |  | x |  |
| 188 | Hysterectomy | x |  |  |  | x | x | x |  | x |  | x | x |
| 189 | Ovarium carcinoma | x |  |  |  | x |  |  |  | x |  | x | x |
| 190 | Ovarium carcinoma | x |  |  |  |  |  |  |  | x |  |  |  |
| 191 | Overactive bladder | x |  |  |  |  |  | x |  | x |  | x |  |
| 196 | Pancreas carcinoma | x |  | x |  | x | x |  |  | x |  |  |  |
| 199 | Parkinson Disease | x |  | x |  | x |  |  |  | x |  |  |  |
| 200 | Parkinson Disease | x |  | x |  |  |  |  |  | x |  | x |  |
| 201 | Parkinson Disease | x |  | x |  |  |  | x |  | x |  | x |  |
| 202 | Carpal Tunnel Syndrome | x |  |  |  | x | x | x |  | x |  | x | x |
| 203 | Periphery Arterial vascular disease | x |  |  |  | x | x | x |  | x |  | x | x |
| 204 | Periphery Arterial vascular disease |  |  |  |  | x |  |  |  | x |  | x |  |
| 205 | Plasklachten man | x |  |  |  | x | x | x |  | x |  | x |  |
| 207 | Pols: gebroken | x | x |  |  | x | x | x |  | x |  | x | x |
| 208 | Ganglion cyst wrist | x |  |  |  | x | x | x |  | x |  | x | x |
| 210 | Keuzehulp: prenatale testen | x |  |  |  | x |  | x |  | x |  | x |  |
| 212 | Pre- en neonatale screeningen (PNS): 20 weken echo | x |  |  |  | x |  | x |  | x |  | x | x |
| 213 | Pre- en neonatale screeningen (PNS): screening op downsyndroom, edwardssyndroom en patausyndroom | x |  |  |  | x | x | x |  | x |  | x | x |
| 214 | Prikkelbare darm syndroom | x |  |  |  | x |  | x |  | x |  | x | x |
| 216 | Keuzehulp voor mannen met gelokaliseerde prostaatkanker | x |  | x |  | x |  |  |  | x |  | x |  |
| 219 | Prostaatkanker keuzehulp | x |  |  |  | x |  | x |  | x |  | x |  |
| 220 | CRPC Keuzehulp | x |  |  |  | x |  | x |  | x |  | x | x |
| 221 | Niet uitgezaaide prostaatkanker (met een hoog risico) | x |  |  |  | x |  | x |  | x |  | x | x |
| 223 | Niet uitgezaaide prostaatkanker (laag risico) | x |  |  |  | x |  | x |  | x |  | x | x |
| 224 | Prostaatkanker | x |  |  |  | x | x | x |  | x |  | x |  |
| 225 | Psoriasis | x |  |  |  | x | x | x |  | x |  | x | x |
| 227 | Psoriasis severe 1 | x |  | x | x | x | x |  |  | x |  |  |  |
| 230 | Reflux | x |  |  |  | x | x | x |  | x |  |  |  |
| 231 | Reflux 2 | x |  |  |  | x | x | x |  | x |  |  |  |
| 232 | Retinaculum cyste | x | x |  |  | x | x | x |  | x |  | x | x |
| 233 | Stoppen met roken | x |  | x | x | x |  |  |  | x |  |  |  |
| 234 | Rotator Cuff: gescheurd | x | x |  |  | x | x | x |  | x |  | x | x |
| 235 | Scafoïd: gebroken | x | x |  |  | x | x | x |  | x |  | x | x |
| 238 | Schouder: gebroken | x | x |  |  | x | x | x |  | x |  | x | x |
| 239 | Schouderinstabiliteit | x |  | x |  | x |  | x |  | x |  | x | x |
| 240 | Sleutelbeen: gebroken aan de buitenkant | x | x |  |  | x | x | x |  | x |  | x | x |
| 241 | Sleutelbeen: gebroken in het midden | x | x |  |  | x | x | x |  | x |  | x | x |
| 243 | Slokdarmkanker: palliatieve behandelopties | x |  |  |  | x |  |  |  | x |  |  |  |
| 244 | Spaakbeenkop: gebroken | x | x |  |  | x | x | x |  | x |  | x | x |
| 248 | Staar | x |  | x |  | x | x |  |  | x |  |  |  |
| 249 | Stressincontinentie | x |  | x |  | x |  | x |  | x |  | x |  |
| 250 | Strottenhoofdkanker keuzehulp | x |  |  |  | x | x | x |  | x |  | x | x |
| 251 | Tenniselleboog | x |  |  |  |  |  | x |  | x |  | x |  |
| 253 | Transmannen: Operatie | x |  | x |  | x | x |  |  | x |  | x |  |
| 254 | Transmannen: vruchtbaarheid | x |  | x |  | x |  |  |  | x |  | x |  |
| 255 | Trauma: herstel na botbreuk | x |  |  |  | x |  | x |  | x |  | x | x |
| 256 | Triggerfinger | x |  |  |  | x | x | x |  | x |  | x | x |
| 257 | Trombose of longembolie | x |  |  |  | x |  |  |  | x |  |  |  |
| 258 | Keuzehulp: weghalen van de baarmoeder (patiënt+) | x |  |  |  | x |  | x |  | x |  | x | x |
| 259 | Uveïtis | x |  | x |  | x |  |  |  | x |  |  |  |
| 260 | Varices | x |  | x |  | x | x | x |  | x |  | x | x |
| 261 | Varices 2 | x |  | x |  | x |  |  |  | x |  |  |  |
| 262 | Veneuze malformaties | x |  | x | x |  |  | x |  | x |  | x |  |
| 263 | Verdoving | x |  | x |  | x | x | x |  | x | x | x |  |
| 264 | Verdoving 2 | x |  |  |  | x | x | x |  | x | x | x |  |
| 266 | Voorstekruisband letsel | x |  | x |  | x | x |  |  | x |  |  |  |
| 267 | Voorstekruisband letsel 2 | x |  |  |  | x | x | x |  | x | x | x |  |
| 268 | Extremely premature birth | x |  |  |  | x | x | x |  | x |  | x | x |
| 271 | Knee arthrosis | x | x |  |  | x | x |  |  |  |  | x | x |
| 274 | Varices |  |  |  |  | x |  |  |  | x |  | x |  |
| 275 | Periphery Arterial vascular disease | x |  | x |  | x | x |  |  | x |  |  |  |
| 276 | Hip arthrosis | x |  |  |  | x | x |  |  | x |  | x | x |
| 277 | Kidney failure | x |  |  |  | x | x |  |  | x |  | x | x |
| 278 | Smoking cessation | x |  |  |  | x | x |  |  | x |  | x | x |
| 280 | Dementia | x | x |  |  | x | x | x |  | x |  |  |  |
| 281 | Prostate carcinoma | x | x |  |  | x |  | x |  | x |  | x |  |
| 282 | Prostate carcinoma | x |  |  |  |  |  |  |  | x |  |  |  |
| 283 | Prostate carcinoma | x |  |  |  | x |  | x |  | x |  | x |  |

1. [↑](#footnote-ref-1)
2. Here answer no if it is not done (so there is numerical information but it has been presented wrongly for example there is no reference class). If there is no numerical information presented answer NA. So if at presenting risk probabilities ‘No was answered’ NA can be answered in the next options [↑](#footnote-ref-2)
